# C-reactive protein and hypertension among Ghanaian migrants and their homeland counterparts: the Research on Obesity and Diabetes among African Migrants study

**DOI:** 10.1097/HJH.0000000000003006

**Published:** 2021-09-02

**Authors:** Joshua A.N. van Apeldoorn, Eva L. van der Linden, Silver Bahendeka, Erik Beune, Karlijn A.C. Meeks, Kerstin Klipstein-Grobusch, Bert-Jan van den Born, Charles Agyemang

**Affiliations:** a Department of Public and Occupational Health, Amsterdam UMC, University of Amsterdam, Amsterdam Public Health Research Institute; b Department of Internal Medicine, Amsterdam UMC, University of Amsterdam, Amsterdam Cardiovascular Sciences, Amsterdam, the Netherlands; c MKPGMS-Uganda Martyrs University, Kampala, Uganda; d Center for Research on Genomics and Global Health, National Human Genome Research Institute, National Institutes of Health, Bethesda, Maryland, USA; e Julius Global Health, Julius Center for Health Sciences and Primary Care, University Medical Center Utrecht, Utrecht University, the Netherlands; f Division of Epidemiology and Biostatistics, School of Public Health, Faculty of Health Sciences, University of the Witwatersrand, Johannesburg, South Africa

**Keywords:** blood pressure, C-reactive protein, Ghanaians, hypertension, inflammation, migrants

## Abstract

**Background:**

Hypertension (HTN) is a growing public health problem in sub-Saharan Africa (SSA) and SSA migrants in Europe. Elevated levels of inflammatory marker C-reactive protein (CRP) have been linked to HTN but the relationship of CRP and HTN among SSA populations has not been studied. To address this knowledge gap, we studied the association between CRP and HTN in migrant and nonmigrant SSA populations residing in different settings.

**Methods:**

Cross-sectional data from the multicentre Research on Obesity and Diabetes among African Migrants (RODAM) study were analysed including 5683 Ghanaians aged at least 18 years, residing in rural and urban Ghana, and Europe. Multivariate logistic regression analyses were used to assess the association between high levels of CRP (≥3 mg/l) and HTN (SBP ≥140 mmHg and/or DBP ≥90 mmHg and/or use of antihypertensive medication) per geographical site and sex.

**Results:**

The association between CRP levels and HTN varied by sex and geographical location. In age-adjusted models, there was an association between high CRP levels and HTN in urban-Ghanaian women (odds ratio 1.50, 95% confidence interval 1.10–2.03), and European-Ghanaian men (1.68, 1.16–2.43) and women (1.63, 1.28–2.07). However, these associations were attenuated after adjustment for conventional risk factors, especially BMI. No association was found in rural-Ghanaians or urban-Ghanaian men.

**Conclusion:**

Our findings show an association between CRP and HTN among Ghanaian migrants and urban-Ghanaian women, however, this was largely explained by conventional risk factors. Thus, prevention of conventional risk factors, in particular obesity, may help to reduce the potentially low-grade inflammatory mechanism underlying HTN.

## INTRODUCTION

Hypertension (HTN) is a growing public health problem in sub-Saharan Africa (SSA) [1,2] and among migrants of SSA origin residing in Europe [3]. SSA, in particular, southern and western Africa, has seen the largest increase in hypertension prevalence compared with other low-income and middle-income regions [4]. SSA migrants residing in Europe are twice as likely to be hypertensive compared with their counterparts residing in SSA [3], and mean blood pressure (BP) levels are higher among SSA migrants than in the European host populations [5]. The reasons for the higher burden of HTN among SSA migrants remain unclear.

A growing body of evidence suggests that inflammation is an important contributor to HTN and HTN-mediated complications [6,7]. C-reactive protein (CRP) is an acute phase protein produced by the liver in response to pro-inflammatory cytokines and is used as a marker for inflammation [8]. CRP is nonspecific and can be elevated secondary to infection, trauma, inflammation, and is also influenced by age and sex [9]. Inflammatory conditions causing raised CRP levels include chronic illness and adiposity [9]. CRP can be elevated secondary to obesity, as visceral adipose tissue produces vast amounts of interleukin-6, which stimulate secretion of CRP by the liver [10]. In pathophysiological studies with genetically modified mice, CRP has been shown to stimulate pro-inflammatory cells, including monocytes. Monocytes activate endothelial cells, reduce production of nitric oxide, and stimulate vascular smooth muscle cell proliferation, resulting in vasoconstriction and subsequent elevating BP [8]. Vice versa, elevated BP might induce a vascular inflammatory state by the continuous high sheer pressure on the vessel wall, resulting in elevated CRP levels [11]. Thus, there appears to be an association between CRP and HTN, although the direction of association remains to be elucidated [12].

With the introduction of high sensitivity CRP (hs-CRP), an assay to accurately measure CRP levels less than 10 mg/l, there has been an increase in human studies assessing the potential role of low-grade inflammation in HTN [7]. Several studies have shown a linear association between CRP concentration and BP, and between high CRP levels and incident HTN [13–16]. However, not all data show a significant association between CRP levels and BP [17,18], and current evidence is insufficient to recommend the measurement of CRP levels to guide decisions in the prevention of, or therapeutic intervention in cardiovascular disease (CVD) [19,20]. Moreover, these studies were primarily conducted among populations of European origin, and there is a paucity of data on this association in SSA populations.

Studying this association between CRP levels and BP in SSA origin populations residing in rural and urban SSA, and European settings may shed light on the potential mechanism driving the high burden of HTN in these populations [3]. It is conceivable that in rural areas in SSA, the population is more exposed to infectious diseases than their urban peers, which might lead to higher CRP levels. Among the SSA populations residing in urban areas in West Africa and among SSA migrants in Europe, obesity is highly prevalent, especially in women, compared with their peers living in rural SSA settings [21]. This high prevalence of obesity may result in CRP elevation [10] in urban areas in SSA and among SSA migrants in Europe. Hence, the underlying causes for CRP elevation may differ among populations living in different locations because of differential exposure to factors influencing inflammation, and hereby potentially having a different impact on BP. Therefore, we examined the association between CRP, BP, and HTN, and assessed whether this association was modified by conventional risk factors among Ghanaians residing in rural and urban areas in Ghana and Ghanaian migrants living in Europe.

## METHODS

### Study population and study design

We used baseline data from the prospective Research on Obesity and Diabetes among African Migrants (RODAM) cohort study. Full details including study rationale, framework, design and methodology have been elaborated on elsewhere [22]. In short, the baseline data collection was highly standardized, multicentred and carried out between 2012 and 2015. The RODAM study included Ghanaians aged at least 18 years living in rural and urban Ghana and in three cities in Europe: Amsterdam, Berlin and London. The different study sites used tailored participant recruitment strategies because of different population registration systems in Europe and Ghana. In brief, in a random sampling procedure, the invited individuals were drawn from enumeration areas in rural and urban Ghana, or municipality registration and member lists of Ghanaian community organizations in Europe. Ethical approval of the study protocols was requested and obtained from the respective ethics committees. Informed written consent was obtained from all study participants before data collection began. Among the invited individuals, the participation rate in rural Ghana was 76 and 74% in urban Ghana. Among the invited individuals in Europe, the participation rates were 75, 68 and 53% in London, Berlin and Amsterdam, respectively. Around 99% of the Ghanaian participants in Europe were first generation migrants.

### Data collection

Data collection was standardized using standard operation procedures in all study sites. Information on sociodemographics, medical history and treatment was obtained by a structured questionnaire [22], either self-administered or via interviews by trained interviewers. Educational attainment was used as proxy of socioeconomic status (SES) and was divided into four categories: none or elementary schooling, lower secondary, higher secondary and tertiary education, or unknown educational level. Physical activity was measured using the WHO Global Physical Activity Questionnaire (GPAQ) version 2 [23]. Afterwards, participants were classified into three categories: low, moderate or high level of physical activity. Current smoking was defined as a positive answer to the question whether the participant smoked [3]. Alcohol intake was classified into three categories: no alcohol intake, alcohol intake according to guideline or more than recommended alcohol intake according to the European Society Cardiology guideline [24] and fruit and vegetable intake in grams per day according to standardized Ghana-specific food propensity questionnaires [25,26]. Participants were categorized as diabetic cases or nondiabetic cases, based on self-report, use of hypoglycaemic medication, fasting blood glucose level of at least 7 mmol/l or a haemoglobin A1c level at least 48 mmol/l, according to the American Diabetes Association criteria [27].

Weight was measured without shoes and in light clothing with SECA 877 scales. Height was assessed without shoes with the SECA 217 stadiometer. BMI was calculated as weight in kilograms divided by height in square metres (kg/m^2^). All anthropometrics of each participant were performed by the same assessor and measured twice; the average of the two measurements was used for analysis. BP was measured after a minimum of 5 min rest and was recorded three times using a semi-automated device (The Microlife WatchBP home) with the use of appropriate cuff size on participant's left arm, with the participant in a seated position. The mean of the last two readings was used in the analysis. HTN was defined as SBP of at least 140 mmHg, or DBP of at least 90 mmHg, or being on antihypertensive medication, based on the participant's prescribed medications list [3].

Fasting venous blood samples were collected by trained research assistants according to standard operating procedures. Shipment procedure and processing of the blood samples have been elaborated elsewhere [22]. High-density lipoprotein (HDL) cholesterol levels and TG levels were determined by using colorimetric test kits and analysed using the ABX Pentra 400 chemistry analyzer (HORBIA ABX, Montpellier, France). High sensitivity CRP (hs-CRP) levels were determined in heparin plasma by a particle-enhanced immunoturbidimetric assay. Human CRP agglutinates with latex particles were coated with monoclonal anti-CRP antibodies. The aggregates were determined turbidimetrically using the ABX Pentra 400 chemistry analyser (HORIBA ABX).

### Statistical analysis

**FIGURE 1:**
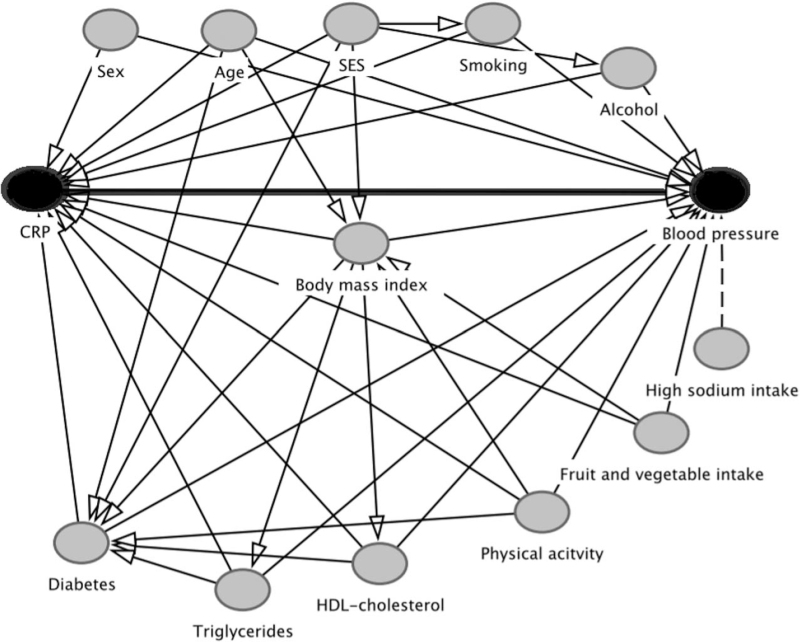
Directed acyclic graph to illustrate the confounding factors in CRP and blood pressure. CRP is the independent variable, blood pressure is the dependent variable. The thick black line indicates the relationship of interest. Straight black lines indicate confounding factors, included in the minimal set of adjustment variables. The dotted line indicates the factor not included in the minimal set of adjustment variables. CRP, C-reactive protein, SES, socioeconomic status, HDL, high-density lipoprotein.

Data were presented as means with standard deviations. Categorical data were presented by frequencies and percentages with 95% confidence interval (CI). Data were stratified by CRP concentrations divided into two levels, normal levels (hs-CRP <3 mg/l) and high levels (hs-CRP ≥3 mg/l) [28]. Analyses were performed for men and women separately because of generally higher CRP levels in women [29] and the large difference in prevalence of being overweight or obese between the sexes in this study population [21]. Chi-square and repeated *t* tests were used to compare prevalence rates for categorical variables, and one-way ANOVA tests to compare means for continuous variables to assess the difference between the groups with normal or high CRP levels. Multivariate logistic regression was used to assess the association between high CRP levels (independent variable) and HTN (dependent variable), with adjustment for covariates. Because of significant interaction between CRP levels and geographical location, defined as Europe, urban and rural Ghana (*P* < 0.001) in the association with HTN, analyses were stratified by geographical location. Multiple linear regression was used to assess the association between high CRP levels and SBP and DBP with adjustments for covariates. A directed acyclic graph (DAG) [30] (Fig. 1) was used to identify minimal sufficient adjustment sets of covariates to determine the unconfounded effect of CRP on HTN and BP variables. Three models were used to examine the data. Model 1 was adjusted for age. Model 2 made additional adjustment for educational attainment as a proxy for SES, as low SES is associated with elevated CRP levels and HTN [31,32]. Model 3 made additional adjustment for BMI, current smoking, alcohol intake, diabetes status, HDL-cholesterol and triglycerides. As CRP levels of more than 10 mg/l have been found to be associated with noncardiovascular causes, such as acute inflammation caused by infections [28], a sensitivity analysis was conducted, excluding participants with hs-CRP levels of greater than 10 mg/l from the regression analyses. Because of positively skewed data, CRP and triglycerides were logarithmically transformed in the linear regression analyses. Two-sided *P* values less than 0.05 were considered statistically significant. Data were analysed using IBM SPSS version 26 for Windows (SPSS Inc, Released 2016; SPSS for Windows, Version 24.0, SPSS Inc, Chicago, Illinois, USA).

## RESULTS

### Characteristics of the study population

**FIGURE 2:**
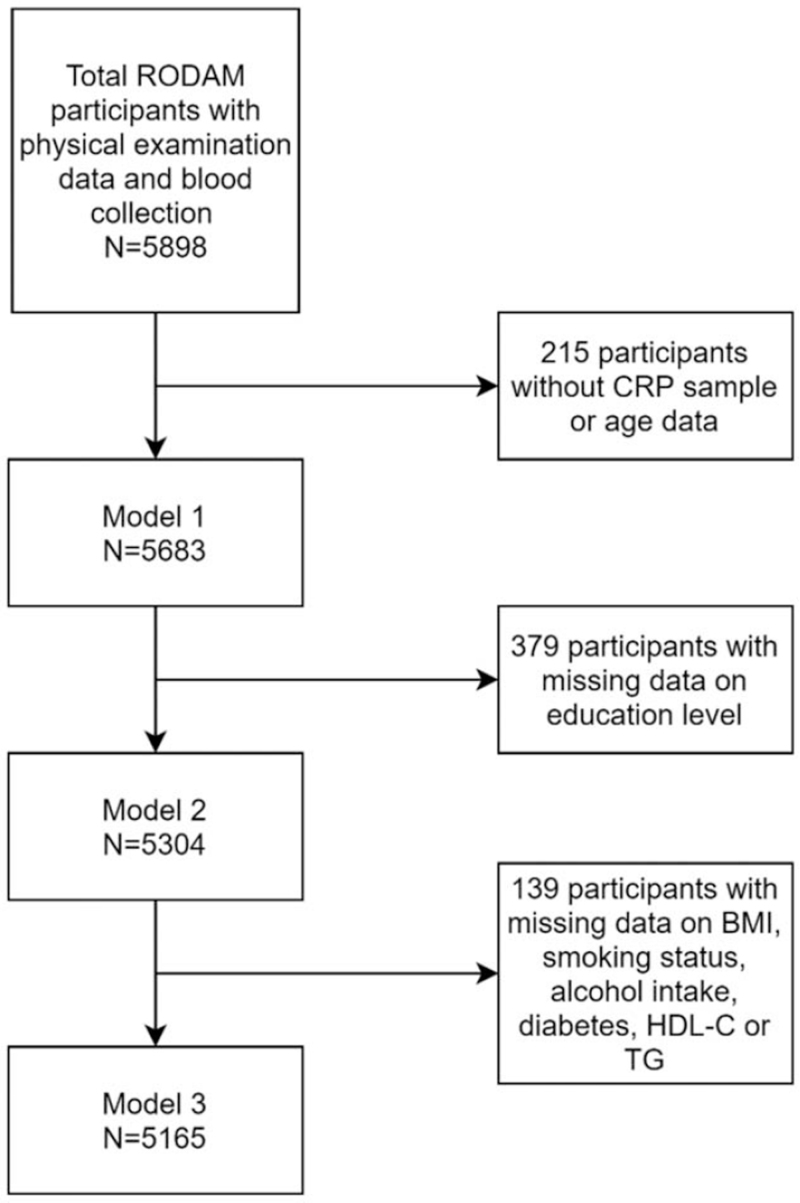
Flow-chart of total excluded Research on Obesity and Diabetes among African Migrants study participants, because of missing data, organized by analysis models. Model 1 was adjusted for age. Model 2 made additional adjustment for education. Model 3 made additional adjustment for BMI, smoking status, alcohol intake, diabetes status, HDL-cholesterol and TG. CRP, C-reactive protein; HDL-C, high-density lipoprotein cholesterol; TG, triglycerides.

A total of 5898 participants who underwent physical examination and blood sample collection were included in the study. After exclusion of participants with missing age values or missing CRP levels (*n* = 215), 5683 participants were included in the analysis (Fig. 2). Of the included participants, 62% were women. Table 1 displays detailed population characteristics of the included participants for men and women separately. Fifteen percent of men and 26.5% of women had high CRP levels. CRP levels varied by geographical location with a greater percentage of participants with high CRP levels in urban and rural Ghana. The prevalence of HTN was high in both CRP groups and both sexes. In women, but not in men, the mean SBP and DBP levels and the prevalence of HTN were significantly higher in the group with high CRP levels than in the group with low CRP levels.

**TABLE 1:** Baseline characteristics

	Men (*n* = 2159)			Women (*n* = 3524)		
	CRP levels less than 3 mg/l	CRP levels at least 3 mg/l	*P* value	CRP levels less than 3 mg/l	CRP levels at least 3 mg/l	*P* value
Number of participants [*n* (%)]	1836 (85.0%)	323 (15.0%)	<0.001	2587 (73.4%)	937 (26.6%)	<0.001
Age [years (SD)]	46.5 (±12.1)	48.5 (±12.5)	0.01	45.1 (±11.6)	47.2 (±11.6)	<0.001
Site [*n* (%)]			0.003			0.001
Amsterdam	513 (86.1%)	83 (13.9%)		681 (74.7%)	231 (25.3%)	
Berlin	268 (86.5%)	42 (13.5%)		219 (81.7%)	49 (18.3%)	
London	365 (89.2%)	44 (10.8%)		484 (74.7%)	164 (25.3%)	
Urban Ghana	348 (83.7%)	68 (16.3%)		720 (69.8%)	310 (30.2%)	
Rural Ghana	342 (79.9%)	86 (20.1%)		483 (72.5%)	183 (28.0%)	
Completed education [*n* (%)]			<0.001			0.43
None or elementary only	343 (81.1%)	80 (18.9%)		1008 (72.0%)	392 (28.0%)	
Lower secondary schooling	676 (83.4%)	135 (16.6%)		848 (74.6%)	288 (25.4%)	
Higher secondary schooling	404 (89.2%)	49 (10.8%)		380 (73.9%)	134 (26.1%)	
Higher vocational schooling, university	293 (89.9%)	33 (10.1%)		182 (75.2%)	60 (24.8%)	
Current smoking status, yes [*n* (%)]	105 (82.0%)	23 (18.0%)	0.17	19 (70.4%)	8 (29.6%)	0.19
Alcohol intake [*n* (%)]			0.56			0.27
No alcohol	1003 (85.7%)	167 (14.3)		1788 (73.6%)	640 (26.4%)	
According to guideline	714 (84.0%)	136 (36.0%)		709 (71.8%)	278 (28.2%)	
More than recommended	99 (85.3%)	17 (14.7%)		45 (80.4%)	11 (19.6%)	
BMI (kg/m^2^) (SD)	25.0 (±4.2)	25.8 (±5.5)	0.03	27.4 (±5.2)	30.6 (±6.3)	<0.001
Diabetes, yes (%)	222 (79.0%)	59 (21.0%)	0.002	190 (54.4%)	159 (45.6%)	<0.001
Triglycerides (mmol/l) (SD)	1.0 (±0.6)	1.2 (±0.6)	0.002	0.9 (±0.5)	1.1 (±0.6)	0.002
HDL-C (mmol/l) (SD)	1.3 (±0.4)	1.2 (±0.4)	<0.001	1.4 (±0.3)	1.3 (±0.4)	<0.001
Hypertension, yes (%)	796 (83.8%)	154 (16.2%)	0.15	948 (68.9%)	427 (31.1%)	<0.001
SBP (mmHg) (SD)	133.4 (±18.7)	134 (±21.1)	0.58	127.5 (±19.0)	131.1 (±20.8)	<0.001
DBP (mmHg) (SD)	83.8 (±12.2)	84.4 (±13.2)	0.56	79.5 (±11.34)	80.8 (±11.71)	0.006

CRP, C-reactive protein; HDL, high-density lipoprotein cholesterol; SD, standard deviation.

### C-reactive protein level and hypertension

Table 2 presents the results of logistic regression analyses for CRP levels and HTN, stratified by site and sex. Compared with their counterparts with CRP levels less than 3 mg/l, Ghanaian men and women residing in Europe and Ghanaian women residing in urban Ghana with CRP levels at least 3 mg/l, had higher odds ratios (OR) for HTN in models 1 and 2. However, this association attenuated after adjustment for conventional risk factors (model 3), particularly after adjustment for BMI and diabetes. Among European-Ghanaian women, high CRP levels persisted to be associated with higher odds for HTN after adjustment for conventional risk factors (model 3). In men in rural and urban Ghana and women residing in rural Ghana, no significant association was found between CRP levels and HTN. In a sensitivity analysis excluding participants with CRP levels greater than greater than 10 mg/l, the association between CRP levels at least 3 mg/l and HTN among European-Ghanaian women was attenuated, whereas the other results remained unchanged (see Table, Supplemental Digital Content 1, which demonstrates this sensitivity analysis).

**TABLE 2:** Odds ratios (95% confidence intervals) for hypertension in participants with high versus low C-reactive protein levels, stratified by site and sex

	*N*	Model 1 OR (95% CI)	Model 2 OR (95% CI)	Model 3 OR (95% CI)
Men				
Europe	1315	1.68 (1.16–2.43)^∗∗^	1.72 (1.16–2.55)^∗^	1.44 (0.95–2.18)
Urban Ghana	416	0.98 (0.54–1.77)	0.92 (0.50–1.71)	0.74 (0.37–1.45)
Rural Ghana	428	0.84 (0.46–1.51)	0.85 (0.46–1.57)	0.79 (0.40–1.58)
Women				
Europe	1828	1.63 (1.28–2.07)^∗∗∗^	1.63 (1.27–2.10)^∗∗∗^	1.34 (1.01–1.76)^∗^
Urban Ghana	1030	1.50 (1.10–2.03)^∗∗^	1.49 (1.09–2.03)^∗^	1.09 (0.78–1.54)
Rural Ghana	666	0.81 (0.54–1.21)	0.85 (0.56–1.30)	0.72 (0.45–1.15)

*N* = participants per site included in model 1. Reference category: CRP level less than 3 mg/l. Model 1: adjusted for age. Model 2: adjusted for age and educational attainment. Model 3: adjusted for age, educational attainment, BMI, smoking, alcohol intake, diabetes, high-density lipoprotein cholesterol and triglycerides. CI, confidence interval; OR, odds ratio.

∗ *P* less than 0.05.

∗∗ *P* less than 0.01.

∗∗∗ *P* less than 0.001.

### C-reactive protein concentration and blood pressure level

Table 3 presents the results of multiple linear regression for logCRP concentration and SBP and DBP separately, stratified by site and sex. Among men and women residing in Europe, higher concentrations of CRP were associated with higher SBP and DBP levels in models 1 and 2; however, this association was attenuated after adjustment for conventional risk factors (model 3). In women residing in urban Ghana, higher CRP concentrations were associated with higher levels of SBP but not with DBP in models 1 and 2. Again, this positive association attenuated in model 3 after adjustment for conventional risk factors. Among men residing in urban Ghana, and among both men and women residing in rural Ghana, no association between CRP concentration and SBP or DBP was observed. Sensitivity analysis excluding participants with CRP levels greater than 10 mg/l in the analysis models did not change the results (see Table, Supplemental Digital Content 2, which demonstrates this sensitivity analysis).

**TABLE 3:** Beta coefficients (95% confidence intervals) for blood pressure levels per 1 unit increase in logarithmically transformed C-reactive protein concentration, stratified by site and sex

	*N*	Model 1 *β* (95% CI)	Model 2 *β* (95% CI)	Model 3 *β* (95% CI)
Men				
Europe				
SBP	1315	2.82 (1.29–4.35)^∗∗∗^	2.69 (1.16–4.21)^∗∗^	1.24 (−0.36–2.84)
DBP	1315	1.58 (0.58–2.57)^∗∗^	1.50 (0.51–2.49)^∗∗^	0.59 (−0.46–1.63)
Urban Ghana				
SBP	416	−0.82 (−3.52–1.88)	−0.82 (−3.53–1.89)	−1.04 (−3.88–1.79)
DBP	416	0.19 (−1.54–1.92)	0.21 (−1.53–1.95)	0.06 (−1.73–1.85)
Rural Ghana				
SBP	428	−1.63 (−4.00–0.74)	−1.71 (−4.11–0.68)	−2.10 (−4.49–0.29)
DBP	428	−0.53 (−2.01–0.96)	−0.60 (−2.10–0.90)	−0.49 (−1.99–1.02)
Women				
Europe				
SBP	1828	2.34 (1.17–3.51)^∗∗∗^	2.33 (1.16–3.50)^∗∗∗^	0.51 (−0.81–1.82)
DBP	1828	1.06 (0.31–1.81)^∗∗^	1.07 (0.315–1.82)^∗∗^	−0.06 (−0.90–0.78)
Urban Ghana				
SBP	1030	3.31 (1.78–4.84)^∗∗∗^	3.32 (1.79–4.85)^∗∗∗^	1.85 (0.15–3.54)
DBP	1030	1.71 (0.75–2.67)	1.71 (0.75–2.67)	0.41 (−0.66–1.47)
Rural Ghana				
SBP	666	0.18 (−1.97–2.34)	0.79 (−1.86–2.44)	−0.34 (−2.61–1.93)
DBP	666	−0.12 (−1.41–1.17)	−0.13 (1.42–1.15)	−0.26 (−1.60–1.09)

*N* = participants per site. Model 1: adjusted for age. Model 2: adjusted for age and educational attainment. Model 3: adjusted for age, educational attainment, BMI, smoking, alcohol intake, diabetes, high-density lipoprotein and logarithmically transformed triglycerides. *β*, beta score; CI confidence interval.

∗ *P* less than 0.05.

∗∗ *P* less than 0.01.

∗∗∗ *P* less than 0.001.

## DISCUSSION

### Key findings

The results of this study show that the association between CRP level and HTN, and between CRP concentration and BP, varied by sex and geographical location of residence. In men and women residing in Europe, and in women residing in urban Ghana, an association was present between CRP and HTN. However, this association was explained by conventional risk factors. In men in urban Ghana, and in rural-Ghanaian men and women, no association was present between CRP and HTN.

### Discussion of the key findings

In age-adjusted and SES-adjusted models, the association between CRP level and HTN, and between CRP concentration and BP appeared to differ between geographical locations. These differences seem to be primarily explained by different underlying factors driving low-grade inflammation, which was in line with our hypothesis. Obesity and diabetes are both more prevalent among Ghanaian migrants residing in Europe and among Ghanaians residing in urban Ghana than among their rural Ghanaian counterparts [21] and appear to be the main factors attenuating the association between CRP and HTN/BP. Our findings are in line with previous results from South Africa [33–35], where CRP was not associated with BP or HTN after adjustment for BMI. As BMI appeared to be correlated with CRP (data not shown), CRP seems to be a risk marker for other CVD risk factors rather than an independent risk factor for HTN [36]. CRP as a possible marker for CVD risk factors is consistent with a Spanish cross sectional study where CRP levels were lower in patients without CVD risk factors who presented with coronary heart disease (CHD), than in CHD patients with CVD risk factors [37]. In rural Ghana, we found no association between CRP and HTN/BP, despite a higher prevalence of increased CRP levels among participants residing in this location. The absence of the association between CRP and HTN/BP in rural Ghana could have several explanations. Firstly, CRP elevation in rural Ghana is more likely because of infectious causes rather than obesity-related inflammation, as in this setting, infectious diseases, such as malaria and helminthic infections, prevail, and risk factors for CVD are relatively less common [38]. To rule out acute infectious causes, which may promote acute and higher CRP elevation [28,39], we excluded participants with CRP levels above 10 mg/l in the sensitivity analysis. However, research has shown that during chronic subclinical infections by malaria and helminthic entities, CRP levels can be within the normal range [40,41]. This suggests that minor CRP elevation because of the permanent higher burden of infectious diseases on the immune system might impact the association between CRP and HTN differently than the chronic inflammatory burden because of conventional cardiovascular risk factors [42,43]. Our results indicate that the cause for CRP elevation needs to be considered before interpreting its relation with HTN, as these differences in cause might affect the impact of CRP on HTN in varying ways. Secondly, differences in lifestyle in rural Ghana compared with the other locations could be underlying the absence of association. For instance, inhabitants of rural Ghana are more physically active and consume more self-grown crops, such as roots, tuber and plantain than inhabitants in urban areas [44]. This high consumption of traditional diet may contribute to the lower prevalence of obesity [21], and thus reducing abdominal visceral fat as a cause for the elevation of CRP levels [10]. Whether the difference in the association between CRP and HTN in rural Ghana and the other study sites is attributable to variation of underlying inflammatory exposures or more optimal lifestyle concerning CVD risk factors, remains to be elucidated.

Another key finding in this study is that in urban Ghana, CRP levels were associated with HTN/BP in women, but not in men in the age-adjusted and sex-adjusted model. This finding of sex differences in the association between CRP and HTN is in line with two previous studies, which found baseline CRP levels to be significantly associated with incident HTN in women [45], but not in men [46]. In urban-Ghanaian women, the association between CRP and HTN disappeared after adjustment for conventional risk factors. This finding is consistent with previous research among a multiethnic cohort, reporting an attenuation of the association between CRP and HTN after adjustment for adiposity in postmenopausal women of African ancestry [47]. Moreover, a multiethnic population-based study showed that an increase in BMI is associated with a much greater increase in CRP levels in women than in men [29], which could explain why high CRP levels were associated with HTN in Ghanaian women but not in men residing in urban Ghana. Another explanation for observed sex differences in associations could be related to hormonal differences. A cross-sectional study that examined the association between CRP levels and HTN in premenopausal, peri-menopausal and postmenopausal women found a nonsignificant, positive association in peri-menopausal and postmenopausal women but a significant positive association in premenopausal women [48], even when adjusting for age and BMI, suggesting the potential role of oestrogen in the sex differences in association.

### Strengths and limitations

Major strengths of the RODAM study include the well standardized data collection in all study sites, and the relatively homogenous nature of the study population (i.e. all Ghanaians with predominantly Akan ethnicity) residing in distinct geographical locations including rural and urban areas in SSA as well as migrant populations in Europe. However, there are also some limitations to our study [22]. Firstly, as in most epidemiologic studies, HTN was based on three measurements in a single occasion. This can be considered a limitation as BP levels may vary along the day, before or after food intake, and white coat HTN cannot be ruled out [49]. Secondly, CRP concentrations could have been affected by active infections. As chronic infections, such as HIV and hepatitis C are known causes of CRP elevations above 10 mg/l in African populations [39], we performed sensitivity analysis by excluding all participants above this CRP threshold. Nevertheless, as previous research has shown that during chronic subclinical infections, CRP levels can be within the normal range [40,41], infections cannot be ruled out completely. However, as the sensitivity analysis excluding participants with CRP levels greater than 10 mg/l did not show large changes in results, except among European-Ghanaian women, we find it unlikely our results to be majorly impacted by acute infection. Thirdly, although CRP has been linked to an increased risk of CVD, such as myocardial infarction, stroke [50,51] and aortic valve stenosis [52], the authors did not assess the relationship between CRP and these CVDs in this study as this was beyond the scope of this work. Furthermore, several conditions that are linked to CRP elevation, such as chronic illnesses [9] were not addressed because of lack of data. Although diabetes was adjusted for in model 3, other concomitant inflammatory or neurodegenerative diseases [53] were not adjusted for, which may possibly influence the association between CRP and HTN. Similarly, social integration could play a role in the association between CRP and HTN. Low social integration is associated with HTN [54] and high social integration is inversely associated with CRP [55]. However, conflicting evidence exist on the association between social integration and inflammation [56], and the reverse association could also be true, that is sickness-induced inflammation could lead to social withdrawal [57]. Therefore, social integration was not included as a variable in the association between CRP and HTN. Fourthly, although physical activity, and fruit and vegetable intake are inversely associated with HTN and CRP [58,59], these variables were not included in model 3, because of the large number of participants with missing values for these variables (20.6%). To check whether these factors impacted the results, the authors performed sensitivity analysis including fruit and vegetable intake and physical activity additionally to model 3, this addition did not change results (data not shown). Lastly, different methods have been used to administer questionnaires. Potentially, this could have influenced how participants answered and could have introduced some level of bias. However, as only 12% of the Ghanaian migrants in Europe self-administered the questionnaire and research assistants were trained to interview in a standardized manner, we consider the potential bias of these different methods limited. Due to the cross-sectional design of this work, conclusions relating causal relationship between CRP and BP should be drawn with caution.

In conclusion, our findings show that the association between CRP levels and HTN in this SSA origin study population varied by sex and geographical location of residence, and that the association was explained by conventional CVD risk factors. Prevention of conventional risk factors, specifically obesity, may help to reduce the potentially inflammatory mechanism underlying HTN in SSA populations. Future research should focus on elucidating potential (inflammatory) mechanism underlying HTN in these SSA populations in different contexts by studying other inflammatory markers and risk factors in order to determine drivers underlying the high burden of HTN.

## ACKNOWLEDGEMENTS

The authors are very grateful to the advisory board members for their valuable support in shaping the methods, to the research assistants, interviewers and other staff of the five research sites, who have taken part in gathering the data and, most of all, to the Ghanaian volunteers participating in this project. We gratefully acknowledge Jan van Straalen from the Academic Medical Centre for his valuable support with standardization of the laboratory procedures and the AMC Biobank for support in biobank management and storage of collected samples.

Financial disclosure: this work was supported by the European Commission under the Framework Programme (Grant Number: 278901). K.A.C.M. is supported by the Intramural Research Program of the National Institutes of Health in the Center for Research on Genomics and Global Health (CRGGH). The CRGGH is supported by the National Human Genome Research Institute, the National Institute of Diabetes and Digestive and Kidney Diseases, the Center for Information Technology and the Office of the Director at the National Institutes of Health (1ZIAHG200362).

### Conflicts of interest

There are no conflicts of interest.

## Supplementary Material

Supplemental Digital Content

## Supplementary Material

Supplemental Digital Content
