# Supplementary material for: C-reactive protein and hypertension among Ghanaian migrants and their homeland counterparts: the Research on Obesity and Diabetes among African Migrants study
Source: J Hypertens. 2021 Sep 2;40(2):283–91. doi: 10.1097/HJH.0000000000003006 (PMC8728758; doi:10.1097/HJH.0000000000003006)
Supplement: Supplemental Digital Content [file jhype-40-283-s001.pdf]

Supplementary table 1 Odds ratios (95% confidence intervals) for hypertension in participants with high versus low C-reactive protein levels, stratified by site and sex, after exclusion of participants with CRP levels >10 mg/L

	N	Model 1<br>OR (95% CI)	Model 2<br>OR (95% CI)	Model 3<br>OR (95% CI)
**Men**				
Europe	1284	1.78 (1.18-2.67)**	1.74 (1.13-2.67)*	1.45 (0.92-2.27)
Urban Ghana	389	1.15 (0.55-2.39)	1.11 (0.52-2.36)	0.83 (0.37-1.89)
Rural Ghana	399	0.84 (0.41-1.71)	0.84 (0.40-1.76)	0.72 (0.30-1.68)
**Women**				
Europe	1732	1.58 (1.21-2.05)**	1.55 (1.18-2.05)**	1.31 (0.97-1.76)
Urban Ghana	946	1.24 (0.88-1.76)	1.22 (0.86-1.74)	0.87 (0.59-1.28)
Rural Ghana	607	0.79 (0.49-1.26)	0.81 (0.50-1.32)	0.59 (0.34-1.02)

N = participants per site included in model 1, OR, Odds ratio, CI, confidence interval

*p<0.05, **p<0.01, ***p<0.001

Reference category: CRP level <3 mg/L

Model 1: adjusted for age

Model 2: adjusted for age and educational attainment

Model 3: adjusted for age, educational attainment, body mass index, smoking, alcohol intake, diabetes, high density lipoprotein and triglycerides
