# Supplementary material for: C-reactive protein and hypertension among Ghanaian migrants and their homeland counterparts: the Research on Obesity and Diabetes among African Migrants study
Source: J Hypertens. 2021 Sep 2;40(2):283–91. doi: 10.1097/HJH.0000000000003006 (PMC8728758; doi:10.1097/HJH.0000000000003006)
Supplement: Supplemental Digital Content [file jhype-40-283-s002.pdf]

Supplementary table 2 Beta coefficients (95% confidence intervals) for blood pressure levels per 1 unit increase in logarithmically transformed C-reactive protein concentration, stratified by site and sex, after exclusion of participants with CRP levels >10 mg/L

		N	Model 1<br>β (95% CI)	Model 2<br>β (95% CI)	Model 3<br>β (95% CI)
**Men**					
Europe	SBP	1284	2.82 (1.29-4.35)***	2.69 (1.16-4.21)**	1.24 (-0.36-2.84)
	DBP	1284	1.58 (0.58-2.57)**	1.50 (0.51-2.49)**	0.59 (-0.46-1.63)
Urban Ghana	SBP	389	-0.82 (-3.52-1.88)	-0.82 (-3.53-1.89)	-1.04 (-3.88-1.79)
	DBP	389	0.19 (-1.54-1.92)	0.21 (-1.53-1.95)	0.06 (-1.73-1.85)
Rural Ghana	SBP	399	-1.63 (-4.00-0.74)	-1.71 (-4.11-0.68)	-2.10 (-4.49-0.29)
	DBP	399	-0.53 (-2.01-0.96)	-0.60 (-2.10-0.90)	-0.49 (-1.99-1.02)
**Women**					
Europe	SBP	1732	2.34 (1.17-3.51)***	2.33 (1.16-3.50)***	0.51 (-0.81-1.82)
	DBP	1732	1.06 (0.31-1.81)**	1.07 (0.315-1.82)**	-0.06 (-0.90-0.78)
Urban Ghana	SBP	946	3.31 (1.78-4.84)***	3.32 (1.79-4.85)***	1.85 (0.15-3.54)
	DBP	946	1.71 (0.75-2.67)	1.71 (0.75-2.67)	0.41 (-0.66-1.47)
Rural Ghana	SBP	607	0.18 (-1.97-2.34)	0.79 (-1.86-2.44)	-0.34 (-2.61-1.93)
	DBP	607	-0.12 (-1.41-1.17)	-0.13 (1.42-1.15)	-0.26 (-1.60-1.09)

N = participants per site, SBP, systolic blood pressure, DBP, diastolic blood pressure, CI confidence interval, β, beta score

*p<0.05, **p<0.01, ***p<0.001

Model 1: adjusted for age

Model 2: adjusted for age and educational attainment

Model 3: adjusted for age, educational attainment, body mass index, smoking, alcohol intake, diabetes, high density lipoprotein and logarithmically transformed triglycerides
